# Phase 1 safety and tolerability study of BMP-7 in symptomatic knee osteoarthritis

**DOI:** 10.1186/1471-2474-11-232

**Published:** 2010-10-10

**Authors:** David J Hunter, Marilyn C Pike, Beth L Jonas, Eugene Kissin, Julie Krop, Tim McAlindon

**Affiliations:** 1 New England Baptist Hospital, Boston. Division of Research, New England Baptist Hospital, 125 Parker Hill Ave, Boston, MA 02120, US; 2 Northern Clinical School, University of Sydney, Sydney, NSW Australia; 3 MedPharm Consulting, Inc (*for Stryker Biotech), Cambridge MA 02138, USA; 4 Thurston Arthritis Research Center, University of North Carolina at Chapel Hill, NC 27514, USA; 5 Rheumatology Department, Boston University Medical Center, Boston MA 02118, USA; 6 Stryker Biotech, Hopkinton, MA 01748, USA; 7 Rheumatology Department, Tufts Medical Center, Boston MA 02111, USA

## Abstract

**Background:**

There are no proven therapies that modify the structural changes associated with osteoarthritis (OA). Preclinical data suggests that intra-articular recombinant human BMP-7 (bone morphogenetic protein-7) has reparative effects on cartilage, as well as on symptoms of joint pain. The objective of this study was to determine the safety and tolerability as well as dose-limiting toxicity and maximal tolerated dose of intra-articular BMP-7. The secondary objectives were to determine the effect on symptomatic responses through 24 weeks.

**Methods:**

This was a Phase 1, double-blind, randomized, multi-center, placebo-controlled, single-dose escalation safety study consisting of 4 dosing cohorts in participants with knee OA. Each cohort was to consist of 8 treated participants, with treatment allocation in a 3:1 active (intra-articular BMP-7) to placebo ratio. Eligible participants were persons with symptomatic radiographic knee OA over the age of 40. The primary objective of this study was to determine the safety and tolerability of BMP-7 including laboratory assessments, immunogenicity data and radiographic assessments. Secondary objectives were to determine the proportion of participants with a 20%, 50%, and 70% improvement in the WOMAC pain and function subscales at 4, 8, 12, and 24 weeks. Other secondary outcomes included the change from baseline to 4, 8, 12, and 24 weeks for the OARSI responder criteria.

**Results:**

The mean age of participants was 60 years and 73% were female. All 33 participants who were enrolled completed the study and most adverse events were mild or moderate and were similar in placebo and BMP-7 groups. The 1 mg BMP-7 group showed a higher frequency of injection site pain and there was no ectopic bone formation seen on plain x-rays. By week 12, most participants in both the BMP-7 and placebo groups experienced a 20% improvement in pain and overall the BMP-7 group was similar to placebo with regard to this measurement. In the participants who received 0.1 mg and 0.3 mg BMP-7, there was a trend toward greater symptomatic improvement than placebo. The other secondary endpoints showed similar trends including the OARSI responder criteria for which the BMP-7 groups had more responders than placebo.

**Conclusions:**

There was no dose limiting toxicity identified in this study. The suggestion of a symptom response, together with the lack of dose limiting toxicity provide further support for the continued development of this product for the treatment of osteoarthritis.

## Background

Recent estimates suggest that symptomatic knee osteoarthritis (OA) occurs in 13% of persons aged 60 and over [1,2]. The risk of mobility disability (defined as needing help walking or climbing stairs) attributable to knee OA alone is greater than that due to any other medical condition in people aged 65 and over [3,4]. Current clinical management for OA is often limited to analgesic medication and cautious waiting [5] for the sometimes eventual referral for total joint replacement. There are no proven therapies that modify the structural changes in osteoarthritis (OA). Given current clinical need and the anticipated increase in prevalence, a treatment that prevents OA progression could have a profound effect on productivity, use of health services and overall quality of life for persons with OA. A number of different intra-articular preparations are in development for potential use in this regard [6-8].

Eptotermin alfa, also called human bone morphogenetic protein-7 (BMP-7), was originally isolated from bone based on its ability to induce new bone formation *in vivo *when combined with an appropriate physical support for matrix cellular attachment.

The recombinant human form of this is referred to as bone morphogenetic protein 7 (BMP-7). BMP-7 is a member of the transforming growth factor (TGF)-β superfamily that contributes to embryonic development and the repair of mature tissues [9]. BMPs signal via transmembrane serine/threonine kinase receptors [10,11]. They can induce a plethora of different cellular effects ranging from stem cell maintenance, migration, differentiation, proliferation to apoptosis. The molecular mechanism, by which the same ligand induces these manifold effects, depends on the cellular context. It is a potent bone-inducing molecule that has been used therapeutically to enhance bone formation in spinal fusions and during fracture repair. In these clinical settings this product has been generally well tolerated.

BMP-7 has also been shown to have reparative effects on cartilage. The biological effects of BMP-7 on cartilage include stimulation of proteoglycan, collagen and hyaluronic acid synthesis; induction of receptors, prevention of catabolism of cartilage components by catabolic factors such as interleukin (IL)-1, but no effect on chondrocyte proliferation and differentiation [12,13].

Compared with normal cartilage, OA cartilage has decreased levels of BMP-7 [14]. OA cartilage explants contain up-regulated receptors for BMP-7, and addition of BMP-7 to human OA cartilage explants results in anabolic activity by increasing extracellular matrix production [15]. Preclinical studies supported this biologic rationale [16-18].

The primary objective of this Phase 1 study was to determine the safety and tolerability as well as dose-limiting toxicity (DLT) and maximal tolerated dose (MTD) of intra-articular BMP-7.

Secondary objectives were to determine the proportion of participants with a 20%, 50%, and 70% improvement in the Western Ontario and McMaster Universities Osteoarthritis Index (WOMAC) pain and function subscales at 4, 8, 12, and 24 weeks

## Methods

This was a double-blind, randomized, placebo-controlled, single-dose escalation safety study consisting of 4 dosing cohorts. Each cohort was to consist of 8 treated participants, with treatment allocation in a 3:1 active to placebo ratio.

The study was conducted at three sites in the US; 2 sites in Boston, Massachusetts and one in Chapel Hill, North Carolina. The local Human Research Ethics Committee at each site approved the study and all participants provided written informed consent. The study was prospectively registered with the NIH Clinical Trials Registry NCT00456157.

### Inclusion Criteria

To be eligible for study participation, a patient had to meet all of the following criteria:

1. Ambulatory persons with OA of the knee with symptoms for at least 6 months and pain on the majority of days in the last 30 days. In participants with bilateral knee OA, the more symptomatic knee was the index knee.

2. Age > 40 years

3. A minimal score of >8 and <16 out of 20 for the WOMAC pain subscale in the index knee at the screening visit after a 2 day NSAID/analgesic washout period. Participants could take acetaminophen up to 1.0 gram every 6 hours.

4. Radiographic evidence on PA and lateral standing, flexed x-rays of at least one osteophyte.

### Exclusion Criteria

1. Concurrent medical or arthritic conditions that could interfere with evaluation of the index knee joint, including fibromyalgia.

2. Participant has received arthroscopic or open surgery to the index joint within 6 months of study start.

3. Corticosteroid, hyaluronic acid or other intraarticular injection within 3 months of study start.

4. Use of chondroitin and/or glucosamine within 4 weeks of study start.

5. History of Reiter's syndrome, rheumatoid arthritis, psoriatic arthritis, ankylosing

spondylitis, lymphoma, arthritis associated with inflammatory bowel disease, sarcoidosis or amyloidosis.

6. Clinical signs and symptoms of active knee infection or crystal disease.

7. Clinically significant cardiac disease.

8. Have an increased predisposition for the development of infections.

9. History of malignancy, with the exception of resected basal cell carcinoma, squamous cell carcinoma of the skin, or resected cervical atypia or carcinoma *in situ*.

10. More significant pain from the back or the hip than the knee.

11. Skin breakdown at the knee where the injection would take place.

12. Planned knee replacement during the study period.

13. For participants undergoing MRI, the presence of contraindications to having an MRI at the specific imaging facility.

14. For participants undergoing MRI, the presence of chronic renal failure defined by a calculated Creatinine Clearance (CrCl) of < 60 mL/min using the Cockcroft-Gault estimate for GFR as follows: CrCl = (140-age [yrs]) × weight [kg]/serum creatinine [mg/dL] × 72 with female gender adjustment (CrCl female = CrCl × 0.85).

15. For participants undergoing MRI, known allergy to gadolinium contrast material.

16. Having known or clinically suspected infection with human immunodeficiency virus (HIV), hepatitis C or B viruses.

17. Having participated within 30 days or will participate concurrently in another investigational drug or vaccine study.

18. Has a history of drug or alcohol dependence in the past 3 years.

19. Known sensitivity to lidocaine or BMP-7.

20. Female with reproductive capability.

21. Prior use of a bone morphogenetic protein.

Participants who met all of the inclusion criteria and none of the exclusion criteria were randomized into the first cohort of 8 participants to receive either 1.0 mL lactose (placebo) (2 participants) or 1.0 mL containing 0.03 mg of BMP-7(6 participants) intraarticularly in an outpatient setting on Day 1. Dose selection was based upon preclinical studies in animals. After an observation period of at least 1 hour, participants were released and contacted via telephone on Day 2 to query for adverse events (AEs) and concomitant medications. Additional follow-up visits were on Days 7, 14, 28, 56, 84 and 168. We did not see participants on Day 2 however all participants were seen by a physician who performed the physical examination at each of the listed visits. After the first and subsequent cohorts of 8 participants each completed through Day 28, the data were reviewed by the Safety Committee (an independent monitor, Principal Investigators from each site and the Study Sponsor) and when deemed appropriate, the next cohort of 8 participants was treated with the next highest dose of BMP-7. The pre-planned dose escalation was 0.03, 0.1, 0.3 and 1.0 mg BMP-7 intra-articularly. Each cohort was randomized in a 3:1 treatment-to-lactose (placebo) ratio.

Randomization to lactose (placebo) or study drug was handled by a call-in randomization process. The investigational pharmacist was unblinded to study drug assignment. Preparation of study drug and lactose (placebo) occurred in the pharmacies which were then transported to the treatment area in 3-mL syringes that appeared identical regardless of whether they contained OP-1 or lactose (placebo).

### Test product, dose and mode of administration

Active Treatment: Recombinant Human BMP-7 (bone morphogenetic protein -7) was supplied as a lyophilized cake in 6-mL vials containing 1 mg. Reconstitution with 1.0 mL sterile water for injection (WFI) resulted in a solution containing 1 mg/mL BMP-7 in 5% lactose (weight/volume). BMP-7 was diluted as needed per dose cohort in 5% lactose.

Lactose (placebo) Control: The lactose (placebo) was provided in a 6-mL vial containing a sterile lyophilized cake of lactose. It was reconstituted with sterile WFI to produce a dosage of 5% lactose in 1.0 mL.

Administration: Given under direct ultrasound or fluoroscopic guidance to ensure injection into the knee joint.

Handling and preparation of BMP-7 drug product and lactose (placebo) were carried out by institution staff in such a way as to maintain study blinding, except that the study pharmacist was unblinded to each participant's study treatment. The institutional pharmacists at each site had access to a code list identifying whether the participant received BMP-7 or lactose (placebo). The pharmacist did not disclose this information to any study personnel. BMP-7 was provided in a single dose 6-mL vial containing a sterile, lyophilized cake of BMP-7.

The participant, study site personnel (other than the research pharmacist), and Stryker Biotech study staff were all blinded to the treatment assignments.

### Safety Assessment

When a minimum of 4 weeks had elapsed since the last participant of the current cohort had been treated, all safety data were collected and reviewed by the safety committee.

Only after the current dose level had been judged to be safe were participants enrolled into the next cohort and dose escalation occurred.

Study sites paid particular heed to the development of any of the following:

• Any evidence of ectopic bone formation not normally associated with OA.

• New or worsening erythema, inflammation and/or joint swelling of the index knee in the first 14 days (2 weeks) following study drug injection.

• The development of an inflammatory infusion in the index knee with WBC > 3,000/mm3 and greater than 30% polymorphonuclear leukocytes.

• Any serious adverse event thought to be at least possibly related to study drug

During the screening period lasting 1 to 28 days, participants provided medical and arthritis history, including the WOMAC questionnaire [19], the KOOS survey[20], SF-36 [21] and VAS global assessments. The WOMAC is a disease specific measure that includes pain (5 items) and physical function (17 items) subscales that are assessed on a 5-point Likert scale for each. The OARSI Standing Committee for Clinical Trials Response Criteria Initiative had developed two sets of responder criteria to present the results of changes after treatment in three symptomatic domains (pain, function, and patient's global assessment) as a single variable for clinical trials with response defined by both a relative and an absolute change [22].

At each visit concomitant medications were recorded, physical examination performed, samples obtained for urinalysis, hematology, chemistry, immunology and serum and plasma banked for biomarker testing. ECG, bilateral knee x-rays was obtained on all participants and an MRI of the index knee in selected participants was done.

At each of the study visits, the following clinical laboratory evaluations were performed:

• Hematology: WBC count, RBC count, hemoglobin, hematocrit, platelet count, WBC differential count.

• Coagulation: PT, aPTT, INR

• Clinical chemistry: Albumin, alkaline phosphatase, ALT (SGPT), AST (SGOT), bicarbonate, bilirubin (total), calcium, phosphorus, chloride, creatinine, glucose, potassium, sodium, total protein, urea (BUN), uric acid.

• Urinalysis: Color, appearance, specific gravity, pH, protein, glucose, ketones, blood, microscopic examination (RBC, WBC, casts, crystals).

At each visit, patient samples were screened for anti-BMP 7 IgM and IgG antibodies. Anti- BMP 7 neutralizing activity was determined only in samples positive for anti- BMP 7 binding antibodies.

Blood samples for serum were obtained just prior to injection, immediately post-injection, 1 hour post-injection and at the 7 day visit to assess BMP 7 drug levels.

Participants were asked to maintain their usual dose of NSAIDs and/or analgesic during the course of the trial, except during the 48 hour period preceding KOOS, SF-36 and patient global assessment evaluations (Acetominophen was permitted during this 48 hour window).

PA and lateral views of the index knee were taken serially in follow-up at Days 28, 84 and 168. At the screening visit, bilateral x-rays were done to determine presence of osteophytes, to determine study eligibility. Follow-up x-rays were performed on the index knee only. X-rays were performed in the 3 recruiting centres and sent for central reading to a musculoskeletal radiologist with osteoarthritis expertise.

Participants enrolled at the Boston sites also had an MRI acquired to assess gadolinium enhanced MRI of their treated knee for dGEMRIC. MRI results were exploratory and given concerns over sample size, adequate powering and also the fact that we did not find anything (either favourable or adverse) these are not further presented due to space limitations.

### Analysis

The primary objective of this study was to determine the safety and tolerability including laboratory assessments, immunogenicity data and radiographic assessments. All participants receiving any part of at least one injection of BMP-7 or lactose (placebo) were evaluated for safety. The safety analyses included evaluation of the incidence of treatment emergent adverse events by preferred term and body system. Laboratory measures were compared with their corresponding normal ranges, and the incidence of abnormally high and of abnormally low laboratory values was calculated for each relevant protocol-specified laboratory test. Fixed flexion PA and lateral x-rays of the index knee for assessment of joint space narrowing, osteophyte changes and ectopic bone formation were performed. As this was primarily a safety study no formal a-priori power calculation was conducted for secondary endpoints of efficacy.

Secondary objectives were to determine the proportion of participants with a 20%, 50%, and 70% improvement in the WOMAC pain and function subscales at 4, 8, 12, and 24 weeks. Other secondary outcomes included the change from baseline to 4, 8, 12, and 24 weeks for the OARSI responder criteria. As this was a Phase 1 study, descriptive statistics (average, standard deviations, minimum, maximum, proportions, and confidence intervals) were used to report study data and no formal statistical techniques have been applied to the data.

## Results

The demographic characteristics of the study sample were broadly consistent between the dosing and placebo cohorts (Table 1). The total study population had a mean age of 59.8 years, a mean BMI of 34.2 kg/m^2 ^and was 73% female. There were no apparent differences in the cohorts recruited from each site. The mean WOMAC pain score (possible range 0-20) was 8.6 for the active treatment groups vs 8.63 for the placebo group. The mean Kellgren and Lawrence Grade at Baseline was 2.8 in the active group vs 3.4 for the placebo group. The mean duration of illness for the placebo group was 9.1 years and active 3.9 years.

**Table 1:** Baseline characteristics of study sample. Mean (SD)

	0.03 mg (N = 7)	0.1 mg (N = 6)	0.3 mg (N = 6)	1 mg (N = 6)	All (N = 25)	Placebo (N = 8)	All Participants (N = 33)
**Age in years**	60.1 (11.2)	58.5 (5.3)	62.2 (15.8)	58.5 (5.6)	59.8 (9.9)	59.8 (6.5)	59.8 (9.1)
**BMI. kg/m^2^**	35.7 (8.7)	35.6 (10.9)	33.0 (7.2)	35.8 (8.2)	35.0 (8.3)	31.6 (5.8)	34.2 (7.9)
**Gender** **(n (%) female)**	3 (43%)	6 (100%)	5 (83%)	4 (67%)	18 (72%)	6 (75%)	24 (73%)

An additional participant was inadvertently recruited into the first 0.03 mg cohort as the notification to the study centers that this cohort was completely recruited occurred after this participant was screened and enrolled.

Most adverse events in the study were categorized as mild or moderate and there were no overall differences in adverse event rates between BMP-7 and placebo groups (Table 2). Two adverse events were categorized as severe and unrelated and occurred in the same participant (0.03 mg group); acute cholecystitis/cholelithiasis (requiring surgery) and vaginal hemorrhage (requiring surgery). There were no life-threatening adverse events and no deaths in the study. There was more injection site pain in the 1 mg BMP-7 group and in the analysis of pain and function, a less durable response in this group. No radiographic abnormalities consistent with ectopic bone formation were reported in follow-up.

**Table 2:** Adverse events by study group

	0.03 mg (N = 7)	0.1 mg (N = 6)	0.3 mg (N = 6)	1 mg (N = 6)	Placebo (N = 8)
**Number of Patients with at least one AE**	7 (100%)	5 (83.3%)	5 (83.3%)	6 (100%)	7 (87.5%)

**Number of Patients with no AE**	0 (0%)	1 (16.7%)	1 (16.7%)	0 (0%)	1 (12.5%)

**Arthralagia**	4 (57.1%)	1 (16.7%)	5 (83.3%)	5 (83.3%)	6 (75%)

**Nasopharyngitis**	2 (28.6%)	2 (33.3%)	1 (16.7%)	0 (0%)	1 (12.5%)

**Upper Respiratory Tract Infection**	1 (14.3%)	0 (0%)	3 (50%)	1 (16.7%)	1 (12.5%)

**Headache**	1 (14.3%)	2 (33.3%)	2 (33.3%)	0 (0%)	1 (12.5%)

**Influenza**	0 (0%)	2 (33.3%)	0 (0%)	0 (0%)	2 (25%)

**Joint Stiffness**	2 (28.6%)	1 (16.7%)	1 (16.7%)	0 (0%)	1 (12.5%)

**Joint Swelling**	2 (28.6%)	0 (0%)	0 (0%)	1 (16.7%)	0 (0%)

**Lymphadenopathy**	0 (0%)	1 (16.7%)	0 (0%)	1 (16.7%)	0 (0%)

**Injection Site Bruising**	0 (0%)	2 (33.3%)	0 (0%)	0 (0%)	0 (0%)

**Injection Site Pain**	1 (14.3%)	0 (0%)	0 (0%)	4 (66.7%)	1 (12.5%)

**Injection Site Mass**	0 (0%)	1 (16.7%)	0 (0%)	1 (16.7%)	0 (0%)

**Injection Site Swelling**	1 (14.3%)	0 (0%)	1 (16.7%)	1 (16.7%)	0 (0%)

Joint swelling occurred in three participants, all were classified as mild and all resolved prior to their final study visit. Two subjects with joint swelling were randomized to the 0.03 dose group, the other to 1.0 mg. Random, minor changes in laboratory values occurred without any apparent trends or clinically significant abnormalities. No patients developed anti- BMP 7 binding antibodies during the study. BMP 7 drug levels results in this study demonstrated that values seen at 1 hr post-dose were very low (near the level of detection of our PK assay) and sporadic, and had no relationship to dose group. Most were non-detectable but a few were near the limit of detection thus not an observable measurement of systemic exposure. There was varied and sporadic usage of rescue medications throughout the study without any clear trends or safety concerns identified. The most common medications administered were ibuprofen and paracetamol.

By 12 weeks most of the participants in the study (both BMP-7 and placebo groups) experienced a 20% improvement in WOMAC pain, and the overall BMP-7 group was similar to placebo (Table 3). In the all BMP-7 group there was a suggestion of a higher proportion of response than for the placebo group for the 50% and 70% reduction endpoints. This trend in the all BMP-7 group was largely driven by effects seen in the 0.1 and 0.3 mg dosing cohorts. These dosing cohorts maintained a trend toward superior 50% improvement and 70% improvement in pain to week 24. The results for WOMAC function are generally consistent with those identified for pain.

**Table 3:** Reduction (improvement) from Baseline in WOMAC Pain. N (%)

		0.03 mg (N = 7)	0.1 mg (N = 6)	0.3 mg (N = 6)	1 mg (N = 6)	All (N = 25)	Placebo (N = 8)
**Mean Baseline Score (0-20)**		7.57	10.5	8.2	8.33	8.6	8.6

**Week 4**	20% Reduction	3 (42.9%)	3 (50.0%)	2 (33.3%)	5 (83.3%)	13 (52%)	1 (12.5%)

**Week 8**	20% Reduction	3 (42.9%)	5 (83.3%)	3 (50.0%)	5 (83.3%)	16 (64%)	3 (37.5%)

**Week 12**	20% Reduction	4 (57.1%)	5 (83.3%)	3(50.0%)	1 (16.7%)	13 (52%)	4 (50.0%)

**Week 24**	20% Reduction	4 (57.1%)	5 (83.3%)	5 (83.3%)	1 (16.7%)	15 (60%)	4 (50.0%)

**Week 4**	50% Reduction	3 (42.9%)	2 (33.3%)	2 (33.3%)	3 (50.0%)	10 (40%)	0 (0.0%)

**Week 8**	50% Reduction	1 (14.3%)	3 (50.0%)	3 (50.0%)	4 (66.7%)	11 (44%)	0 (0.0%)

**Week 12**	50% Reduction	0 (0.0%)	3 (50.0%)	2 (33.3%)	1 (16.7%)	6 (24%)	1(12.5%)

**Week 24**	50% Reduction	0 (0.0%)	5 (83.3%)	3 (50.0%)	1 (16.7%)	9 (36%)	0 (0.0%)

**Week 4**	70% Reduction	1 (14.3%)	1 (16.7%)	2 (33.3%)	1 (16.7%)	5 (20%)	0 (0.0%)

**Week 8**	70% Reduction	1 (14.3%)	1 (16.7%)	2 (33.3%)	1 (16.7%)	5 (20%)	0 (0.0%)

**Week 12**	70% Reduction	0 (0.0%)	2 (33.3%)	2 (33.3%)	0 (0.0%)	4 (16%)	0 (0.0%)

**Week 24**	70% Reduction	0 (0.0%)	3 (50.0%)	3 (50.0%)	0 (0.0%)	6 (24%)	0 (0.0%)

The OARSI responder criteria analysis suggested that there were more responders in the BMP-7 groups than in the placebo (Table 4). The greatest response was again seen in the 0.1 and 0.3 mg dosing cohorts.

**Table 4:** OARSI responder criteria

Number (%) of Responders	0.03 mg (N = 7)	0.1 mg (N = 6)	0.3 mg (N = 6)	1 mg (N = 6)	All (N = 25)	Placebo (N = 8)
**Week 4**	3 (42.9%)	4 (66.7%)	1 (16.7%)	2 (33.3%)	10 (40%)	1 (12.5%)
**Week 8**	3 (42.9%)	4 (66.7%)	2 (33.3%)	2 (33.3%)	11 (44%)	0 (0%)
**Week 12**	1 (14.3%)	4 (66.7%)	2 (33.3%)	1 (16.7%)	8 (32%)	1 (12.5%)
**Week 24**	1 (14.3%)	5 (83.3%)	2 (33.3%)	1 (20%)	9 (37.5%)	1 (12.5%)

## Discussion

The results of this Phase 1 study are reassuring for further drug development. There was no dose limiting toxicity identified in this study overall and no differences in adverse event rates between BMP-7 and placebo groups. There was however more injection site pain reported in the 1 mg BMP-7 group and a less durable response in pain and function improvement in this specific group. Importantly there was no evidence of ectopic bone formation on serial radiographic evaluation.

The trend for BMP-7 symptom response was better than placebo with the 0.1 and 0.3 mg groups. The bell shaped dose response curve is consistent with pharmacodynamic studies in pre-clinical models (data not shown). At this early stage of development the signal identified in this study is reassuring as similar small trials of intra-articular therapies for OA (such as IL-1 inhibitors) have not had similar early phase success to encourage further development [6]. Similarly the trends seen in the 1 mg dose group of increased injection site pain and less apparent efficacy would suggest this dose not be pursued in further studies. Preclinical data are roughly equivalent in pattern compared to the reported clinical results; i.e. as expected, there are low doses that are ineffective, higher doses that scale to a similar dose in humans that are effective and very high doses that are either less effective or ineffective. A non-linear dose response is not atypical with biologic therapy.

One dose administration was selected as with many growth factors, there is no clear linkage between the pharmacodynamic effects of BMP-7 and its temporal presence in the blood or at the site of action. BMP dimers bind to specific receptors on the surface of mesenchymal stem cells and initiate a cascade of events that eventually leads to the differentiation of new tissues in the following weeks and months. Preclinical evidence suggests that a single injection/treatment of BMP-7 is sufficient to trigger a cascade of bone formation from months to years.

There are a number of limitations of the current study that warrant further description. This was primarily a dose-finding safety study and hence the number of participants is small. Any suggestion of efficacy signals for pain or function need to be adequately determined in appropriately powered clinical trial/s.

The imaging assessments conducted as part of this study provided no suggestion of either an adverse safety profile or hints of efficacy. We do know (via unpublished preclinical data) that there is the potential for differentation of stem cells to the osteogenic lineage under BMP-7 stimulation. The only consistent observation among the various IA preclinical studies was the sporadic formation of small foci (generally no larger than 1-5 mm in diameter) of bone along the needle track between the skin and the outside of the joint capsule. Such foci are likely the end result of residual product at the needle tip. In our human study, the 6 month x-rays were evaluated for both osteophytes changes and evidence of ectopic bone formation. No patients had any evidence of ectopic bone formation at six months. To fully understand the structural effects of BMP-7 administration in OA will also require adequately powered clinical trials following participants for 1-2 years.

2 participants were reported to have lymphadenopathy during follow-up. In preclinical animal models, there is no data that are suggestive of lymphadenopathy in any species at does many fold the dose used in our clinical trials. Additionally, we see no evidence of meaningful changes in circulating white blood cell counts.

The success of agents that ultimately seek regulatory approval for disease modification in OA will be critically dependent upon the demonstration of symptom improvement. Consistent with this need BMP-7 appears to demonstrate an early signal for symptom improvement that may facilitate seeking a pain indication followed by structural modification claims.

## Conclusions

This trend to a symptom response, together with the lack of dose limiting toxicity provide further support for the continued development of this product for treatment of osteoarthritis. The injection site pain adverse event profile at 1.0 mg coupled with the promising symptom response will likely see further pursuit of dosing groups consistent with 0.1 and 0.3 mg dosing cohorts in Phase 2.

## List of abbreviations

AE: adverse event; BMI: body mass index; BMP: bone morphogenetic protein; dGEMRIC: delayed Gadolinium Enhanced MRI of Cartilage; DLT: dose-limiting toxicity; IL-Interleukin; KOOS: Knee osteoarthritis outcome score; MTD: maximal tolerated dose; OA: osteoarthritis; OARSI-Osteoarthritis Research Society International; PA: posteroanterior; SD: standard deviation; TGF: transforming growth factor; VAS-visual analogue scale; WOMAC: Western Ontario and McMaster Universities Osteoarthritis Index

## Competing interests

The corresponding author had full access to all the data in the study and had final responsibility for the decision to submit for publication. MP received consulting fees from Stryker Biotech from April 2005 through December 2008 through MedPharm Consulting, Inc. The consulting involved trial design, protocol development, study initiation, medical and safety monitoring, final data review and Clinical Study Report preparation for this Phase I trial. Annual reports were also prepared for submission to regulatory agencies. EK received salary support from UCB during the time of the study. JK works for Stryker and holds stock options for Stryker. There are currently patents pending on the use of BMP-7 in OA.

## Authors' contributions

DJH participated in the design and coordination of the study and drafted the manuscript.

MCP and JK participated in the design and coordination of the study. BLJ, EK, and TM participate as site investigators and collected data. All authors read and approved the final manuscript.

## Pre-publication history

The pre-publication history for this paper can be accessed here:

http://www.biomedcentral.com/1471-2474/11/232/prepub
